# A *Francisella*-like endosymbiont in the Gulf Coast tick evolved from a mammalian pathogen

**DOI:** 10.1038/srep33670

**Published:** 2016-09-20

**Authors:** Jonathan G. Gerhart, Abraham S. Moses, Rahul Raghavan

**Affiliations:** 1 Department of Biology and Center for Life in Extreme Environments, Portland State University, Portland, Oregon, 97201, USA

## Abstract

Ticks (order Ixodida) vector pathogenic bacteria that cause diseases in humans and other mammals. They also contain bacteria that are closely related to pathogens but function as endosymbionts that provide nutrients that are missing from mammalian blood—their sole food source. For instance, mammalian pathogens such as *Coxiella burnetii* and *Francisella tularensis*, as well as *Coxiella*-like and *Francisella-*like endosymbionts (CLEs and FLEs, respectively) occur in ticks worldwide. However, it is not clear whether the pathogens evolved from symbionts or symbionts from pathogens. Recent studies have indicated that *C. burnetii* likely originated from a tick-associated ancestor, but the origins of FLEs are not clear. In this study, we sequenced the genome of an FLE, termed FLE-Am, present in the Gulf Coast tick, *Amblyomma maculatum*. We show that FLE-Am likely evolved from a pathogenic strain of *Francisella*, indicating that tick endosymbionts can evolve from mammalian pathogens. Although the genome of FLE-Am is almost the same size as the genomes of pathogenic *Francisella* strains, about one-third of its protein-coding genes contain inactivating mutations. The relatively low coding capacity and extensive metabolic capabilities indicate that FLE-Am transitioned recently to its current endosymbiotic lifestyle and likely replaced an ancient endosymbiont with degraded functionality.

Blood is the sole source of nutrition for ticks; however, it contains minimal amounts of several amino acids and cofactors required for normal development^1^. Similar to insects that thrive on unbalanced plant diets^2^, ticks depend on symbiotic bacteria such as *Coxiella*-like and *Francisella*-like endosymbionts (CLE and FLE, respectively) that likely provision nutrients missing from their diet^3^,^4^. In addition to containing endosymbionts, ticks often vector closely related mammalian pathogens, including *Francisella tularensis* and *Coxiella burnetii*^5^,^6^, but the evolutionary relationship between the two clades is not clear. Among *Amblyomma* species (hard-backed tick, family Ixodidae), the most prevalent endosymbionts are CLEs, which have reduced genomes, a feature associated with long-term endosymbiosis^7^. Interestingly, unlike most other *Amblyomma* species, the primary endosymbiont of *A. maculatum* (Gulf Coast tick) is a FLE, termed FLE-Am^8^. We sequenced its genome and show that it likely evolved from a pathogenic ancestor, thereby showing that tick endosymbionts could evolve from mammalian pathogens. Unlike the highly reduced genomes of CLEs, FLE-Am has undergone minimal genome reduction, but a substantial number of its protein-coding genes, including virulence genes, contain inactivating mutations. This low coding capacity indicates that the endosymbiont originated recently, and additionally, by virtue of its superior metabolic and biosynthetic capabilities, FLE-Am most likely replaced an ancestral CLE with degraded functionality, thereby allowing *A. maculatum* to escape the “symbiosis rabbit hole”^9^.

## Results and Discussion

### FLE-AM evolved from a pathogenic ancestor

**Figure 1:**
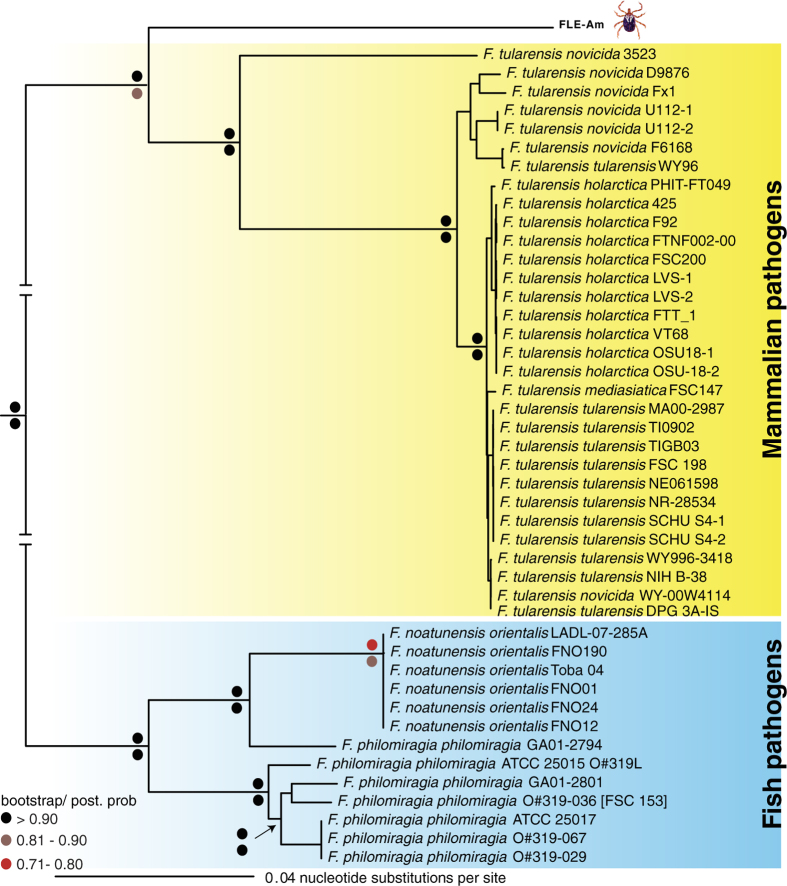
FLE-Am is a sister taxon of mammalian pathogens. Phylogenetic tree based on 442 orthologous genes in FLE-Am and 44 fully sequenced *Francisella* genomes is shown. Bootstrap and posterior probability values are provided on top and bottom of each node, respectively.

We analyzed the *A. maculatum* microbiome and discovered that FLE-Am dominates the tick microbiome. Out of the 4,603 (>1 kb) contigs assembled from the sequencing reads, a vast majority was of host origin. Only 48 contigs were of bacterial origin, and out of those 46 belonged to Francisellacae (Supplementary Table S1). FLE-Am has been previously shown to be the major bacterium present in *A. maculatum*^8^, and FLEs are maternally transmitted^10^, indicating that it is essential to the normal development of *A. maculatum*. We estimated the phylogenetic position of FLE-Am using 442 orthologous proteins present in 44 fully sequenced *Francisella* genomes. As shown in Fig. 1, FLE-Am shares a recent common ancestor with *Francisella* species that are pathogenic to mammals, with the exclusion of aquatic *Francisella* species that are pathogenic to fish. Our multi-protein tree clarifies earlier phylogenetic trees based on partial FLE-Am 16S rRNA sequences that showed it to be a sister taxon of *F. tularensis*^11^.

### Virulence genes have been inactivated in FLE-AM

**Figure 2:**
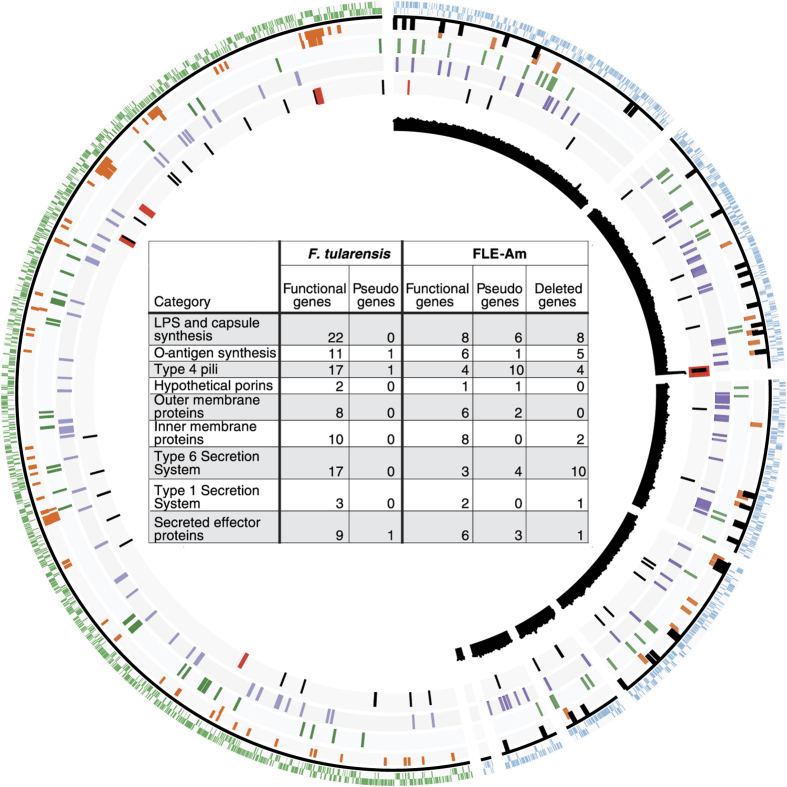
Comparison of FLE-Am and *F. tularensis* genomes. FLE-Am genome is on the right and *F. tularensis tularensis* SCHU-S4 genome is on the left. The outer two rings show protein-coding genes (blue in FLE-Am and green in *F. tularensis*). Rings 3 and 4 contain virulence genes (intact genes in orange and pseudogenes in black). Amino acid biosynthesis genes (green) are in ring 5. Cofactor synthesis and transport genes (purple) are in ring 6, and tRNAs (black) and rRNAs (red) are in ring 7. Sequencing read coverage for FLE-Am is shown in the innermost black semi-circle. Notice that the coverage for the rRNA operon is double that of the rest of the genome. The table in the center highlights the inactivation and loss of virulence genes in FLE-Am.

As shown recently for *C. burnetii*, mammalian pathogens could evolve from non-pathogenic ancestors by acquiring virulence genes^12^; conversely, avirulent symbionts could arise from pathogenic ancestors by losing virulence genes, but no clear examples of this process have been documented. To identify the evolutionary relationship between pathogenic *Francisella* and FLE-Am, we examined the FLE-Am genome for the presence of virulence genes described in *F. tularensis* and *F. novicida*^13^,^14^. We discovered that FLE-Am contained pseudogenized versions of several virulence genes, including genes for a Type VI Secretion System present on a pathogenicity island in *F. tularensis* and for Type 4 pili that are critical to mammalian infection (Fig. 2, Supplementary Table S2). Collectively, our data denotes that the ancestor of FLE-Am was most likely a mammalian pathogen that contained functional versions of virulence genes. The absence of intact secretion and effector gene systems in FLE-Am suggests that it is avirulent to humans despite its presence in the salivary glands and saliva of *A. maculatum*^8^. Intriguingly, salivary glands of other *Amblyomma* species such as *A. americanum* contain CLEs^15^. Although its functional significance is not understood, being secreted in saliva could facilitate the exchange of endosymbionts between ticks while co-feeding on the same host^16^.

### FLE-Am evolved recently and likely replaced an ancestral endosymbiont

The genome of FLE-Am (1.56 Mb) is ~80% the size of the genome of the mammalian pathogen *F. tularensis* (1.89 Mb) (Table 1), and a significant portion (~33%) of its protein-coding genes contains inactivating mutations (Fig. 2, Supplementary Dataset 1). The degree of reductive genome evolution in FLE-Am is much lower than what is usually observed in long-term endosymbionts such as a CLE in *A. americanum* (CLEAA)^3^, and the presence of large number of pseudogenes imply that the bacterium is in the initial stages of reductive evolution, as superfluous genes are first pseudogenized and then ultimately deleted from the genome when a bacterium shifts from a free-living to a host-associated lifestyle. Additionally, endosymbiont genomes tend to be much more A+T biased than closely related pathogens or environmental bacteria; however, the nucleotide composition (G+C%) of FLE-Am is very similar to that of *F. tularensis* (32. 3% and 31.8%, respectively)^7^,^9^. These features collectively indicate that FLE-Am transformed recently into an endosymbiont.

**Figure 3:**
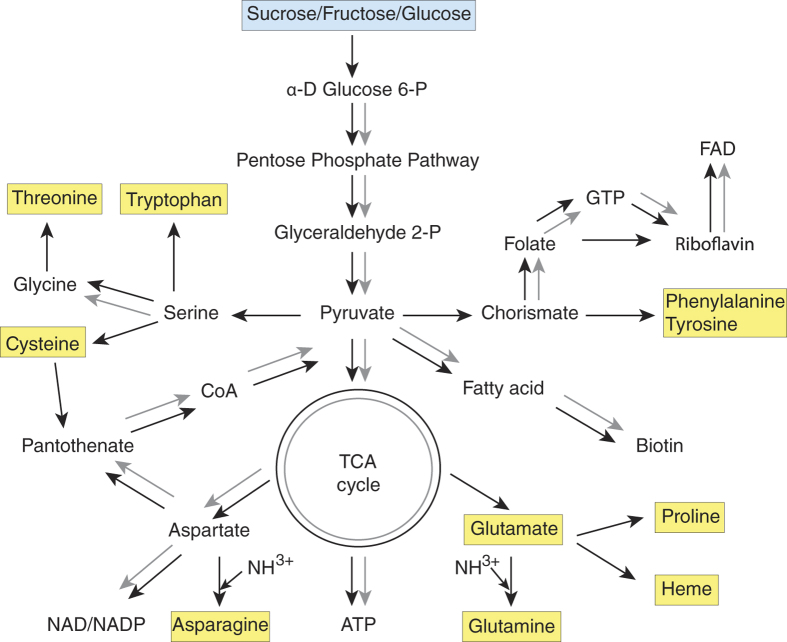
Comparison of metabolic capabilities of FLE-Am and CLEAA. Black arrows mark pathways present in FLE-Am, and grey arrows depict CLEAA pathways. Metabolic products exclusively produced by FLE-Am are highlighted in yellow.

Because bacteria with extensive metabolic proficiency are known to replace ancient endosymbionts with reduced metabolic prowess^9^, we compared the metabolic pathways present in FLE-Am to that of CLEAA, the cofactor provisioning endosymbiont found in *A. americanum*^3^. As shown in Fig. 3, the metabolic capability of FLE-Am is much more extensive than in CLEAA. For instance, FLE-Am can produce heme in addition to cofactors (except thiamine) synthesized by CLEAA. Furthermore, while both FLE-Am and CLEAA share the ability to produce aspartate from pyruvate, and to metabolize it into ATP, only the FLE synthesizes cysteine, threonine, tyrosine, tryptophan, phenylalanine, and serine from pyruvate, and can metabolize glutamate, glutamine, and asparagine into ATP. Of these amino acids, FLE most crucially provides a reliable source of the amino acid cysteine, which is found in very low concentrations in bovine blood^1^. FLE-Am can also synthesize glutamine from glutamic acid and ammonia, thus recycling cellular ammonia waste to useful products. In sum, our data indicate that the superior biosynthetic capability of FLE-Am confers a selective advantage, which could have led to FLE-Am recently replacing an ancestral symbiont (e.g. CLE) with reduced metabolic capacity in *A. maculatum*.

**Figure 4:**
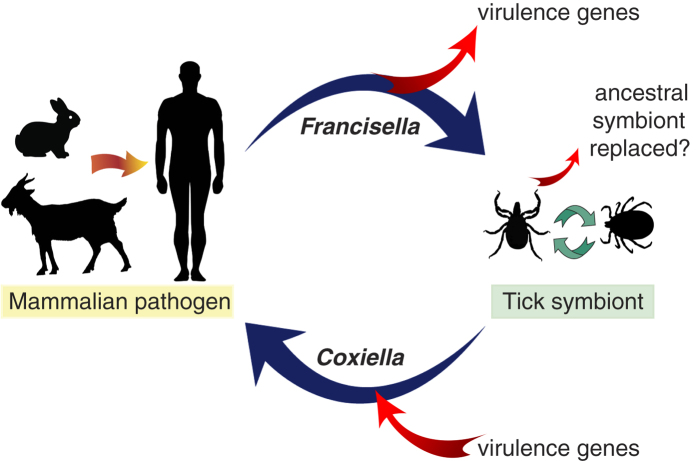
Evolution of mammalian pathogens and tick symbionts. Mammalian pathogens could evolve from tick-associated bacteria by acquiring virulence genes (e.g., *Coxiella burnetii*). Conversely, evolution of tick endosymbionts from mammalian pathogens is associated with loss of virulence genes (e.g., FLE-Am).

Symbiosis with bacteria allows arthropods to thrive on nutrient-poor diets^2^. However, the absolute reliance on a symbiont could eventually become disadvantageous to the host because massive gene loss and accumulation of mildly deleterious mutations will corrode the symbiont’s metabolic capability^7^,^9^. One solution to escaping this symbiosis “rabbit hole” is to supplement or replace the ancient symbiont with new bacteria acquired from the environment^9^. This process has occurred in several lineages of insects (e.g. ref. 17). In *A. maculatum*, FLE-Am seems to have recently replaced an older endosymbiont, whose identity is unknown, but based on the current bacterial prevalence data it was most probably a CLE or a *Rickettsia*^12^. FLE-Am likely arose by the domestication of a mammalian pathogen that was vectored by the tick. This process could occur rapidly because the bacterium doesn’t have to learn anew how to circumvent the tick’s immune response, and there is no need to attenuate bacterial virulence towards the tick^18^. Additionally, because genes that promote pathogenesis through amino acid scavenging (e.g., FTT 0968c, *xasA*), macrophage survival (e.g., *carA, carB*, *bioF*), and intracellular replication (*aroE1*, *purMCD*, *purL*, and *purF*)^13^,^14^ are retained in FLE-Am (Fig. 1), they could be key to its endosymbiotic lifestyle. Further evolutionary, functional and genomic studies of FLEs and CLEs from a wide array of soft and hard ticks will help us to better understand how pathogenic *Francisella* evolved into endosymbionts that supports the blood-dependent lifestyle of ticks, and how tick-associated *Coxiella* evolved into pathogenic *C. burnetii*^12^ (Fig. 4).

## Methods

### Sequencing and bacterial identification

DNA was extracted from a female *A. maculatum* procured from Oklahoma State University Tick Rearing Facility, as described previously^3^, and was sequenced using Illumina Hi-Seq 2500 (100 cycles, paired-end) at OHSU MPSSR, yielding approximately 180 million read pairs. Low confidence reads were removed, and the identify of bacteria present in the tick microbiome were determined utilizing ≥1 kb contigs binned using MEGAN^19^.

### Genome Assembly

Trimmed reads were assembled into contigs using IDBA-UD^20^, and by comparing them to *Francisella* genome sequences, FLE contigs were identified. All trimmed reads were mapped to these contigs and then reassembled with IDBA-UD and IDBA-hybrid into a final set of seven contigs (Supplementary Table S3), which were submitted to NCBI (Accession: LVCE00000000). The completeness of the assembled genome was examined using a single-copy gene database^21^, and as a control, an identical single-copy gene analysis was performed on *F. tularensis subsp. tularensis* SCHU S4 genome (NC_006570.2) (Table 1).

### Genome Annotation

FLE-Am contigs were annotated using NCBI Prokaryotic Genome Annotation Pipeline, and pseudogenes were verified manually. Protein-coding genes were binned into categories based on their role in primary metabolism, amino acid and nucleic acid synthesis, or vitamin and cofactor metabolism, and then subsequently compared to CLEAA to illustrate differences in their metabolic capabilities. A list of genes critical to the pathogenicity of *F. tularensis*^13^,^14^ was used to identify both functional and pseudogenized versions of virulence genes present in FLE-Am (Supplementary Table S2). Intact protein-coding genes present in FLE-Am but not in *F. tularensis* SCHU-S4 are provided in Supplementary Table S4.

### Phylogenetic Analysis

In addition to FLE-Am, we included all 44 fully sequenced *Francisella* genomes to generate a robust phylogenetic tree. Using reciprocal BLASTP, a subset of 442 orthologous genes (Supplementary Dataset 2) that were conserved in all genomes was identified. Nucleotide sequences were aligned using Clustal Omega^22^, ambiguously aligned regions were removed using Gblocks^23^, and jModelTest2 was used to select the GTR+I+G (General Time Reversible plus Invariant sites plus Gamma distribution) evolution model^24^. Using concatenated sequences, Maximum Likelihood trees with 1,000 bootstrap replicates were constructed using RAxML^25^. Bayesian trees with a chain length of 500,000 and a burn-in fraction of 25% and sampling every 100 trees were constructed using MrBayes^26^.

## Additional Information

**How to cite this article**: Gerhart, J. G. *et al*. A *Francisella*-like endosymbiont in the Gulf Coast tick evolved from a mammalian pathogen. *Sci. Rep.* **6**, 33670; doi: 10.1038/srep33670 (2016).

## Supplementary Material

Supplementary tables

Supplementary Dataset 1

Supplementary Dataset 2

## Figures and Tables

**Table 1:** Genome features of FLE-Am and *F. tularensis* SCHU-S4.

	*F. tularensis*	FLE-Am
Genome size (bp)	1,892,772	1,556,255
GC%	32.3	31.8
Protein-coding genes	1,556	1,001
Pseudogenes	227	484
rRNAs	10	7
tRNAs	38	32
Single copy genes	106/111	106/111
Average Gene Size	942 bp	886 bp
GenBank Accession	NC_006570.2	LNCT00000000
