# Supplementary material for: A Francisella-like endosymbiont in the Gulf Coast tick evolved from a mammalian pathogen
Source: Sci Rep. 2016 Sep 20;6:33670. doi: 10.1038/srep33670 (PMC5028885; doi:10.1038/srep33670)
Supplement: Supplementary tables [file srep33670-s1.pdf]

A *Francisella*-like endosymbiont in the Gulf Coast tick evolved from a mammalian pathogen

Jonathan G. Gerhart, Abraham S. Moses, Rahul Raghavan

Department of Biology and Center for Life in Extreme Environments,

Portland State University, Portland, Oregon, 97201, USA.

Table S1. Assembly statistics for FLE-Am contigs.

Identity	Contigs >1kb	Total Length (bp)	Reads	% GC
Trimmed Reads			291,344,244	45
Assembled Contigs	4603	9,312,626	10,274,914	43
Bacteria Assigned	48	1,560,742	1,287,780	33
Francisellacae	46	1,614,058	1,259,156	32.90
Flavobacteriaceae	1	2,366	908	38.10
Anaeromyxobacter	1	2,121	1,194	59.40
N50 (386,665 bp)				

Table S2. Pathogenic features in FLE-Am and *F. tularensis* SCHU-S4.

Category[a]	Locus tag[b]	Function	*F. tularensis* SCHU-S4	FLE-Am[c]
**LPS, Capsule and Membrane synthesis**				
	FTT_0286c	LPS	+	Φ
	FTT_0454	LPS	+	Φ
	FTT_0455c	LPS	+	+
	FTT_0789	Capsule	+	+
	FTT_0790	Capsule	+	Φ
	FTT_0791	Capsule	+	Φ
	FTT_0792	Capsule	+	-
	FTT_0793	Capsule	+	-
	FTT_0794	Capsule	+	-
	FTT_0795	Capsule	+	-
	FTT_0796	Capsule	+	-
	FTT_0797	Capsule	+	-
	FTT_0798	Capsule	+	-
	FTT_0799	Capsule	+	-
	FTT_0800	Capsule	+	Φ
	FTT_0805	Unkown	+	+
	FTT_0807	Unkown	+	+
	FTT_0806	Unkown	+	Φ
	FTT_0891	LPS	+	+
	FTT_1236	Capsule	+	+
	FTT_1237	LPS	+	+
	FTT_1238c	Capsule	+	+
	FTT_1450c	Both	+	Φ
	FTT_1453c	LPS	+	-
	FTT_1455c	Both	+	-
	FTT_1458c	LPS	+	-
	FTT_1460c	LPS	+	-
	FTT_1461c	LPS	+	-
	FTT_1462c	Both	+	+
	FTT_1463c	Both	+	+
	FTT_1464c	Both	+	+
	FTT_1561	LPS	+	+
	FTT_1571c	LPS	+	+
	FTT_1643c	LPS	Φ	+
**Type 4 Pili**				
	FTT_1134	Pilin	+	Φ
	FTT_0890c	Pilin	+	Φ
	FTT_0889c	Pilin	+	Φ
	FTT_0888c	Pilin	+	Φ

	FTT_0861c	Pilin	+	Φ
	FTT_0230c	Pilin	+	+
	FTT_1341c	Pilin	+	+
	FTT_1133	Assembly ATPase	+	Φ
	FTT_0905	PilE1 glycosylase	+	Φ
	FTT_1156c	OM unit	+	Φ
	FTT_0088	Retraction ATPase	+	Φ
	FTT_0715	Secreted	+	-
	FTT_1786c	Secreted	+	-
	FTT_1577	Secreted	+	Φ
	FTN_1186	Secreted	-	-
	FTT_1069	Secreted	+	-
	FTT_0580	Secreted	+	+
	FTT_1330	Secreted	+	+
**Hypothetical porins**				
	FTT_0025c	Outer Membrane	+	Φ
	FTT_0119	Outer Membrane	+	+
**Outer Membrane Proteins**				
	FTT_0369c	Outer Membrane	+	+
	FTT_0583	Outer Membrane	+	+
	FTT_0831c	Outer Membrane	+	+
	FTT_0918	Outer Membrane	+	+
	FTT_1103	Outer Membrane	+	+
	FTT_1346	Outer Membrane	+	Φ
	FTT_1416c	Outer Membrane	+	Φ
	FTT_1724c	Outer Membrane	+	+
**Inner Membrane Proteins**				
	FTT_0094c	Inner Membrane	+	+
	FTT_0181c	Inner Membrane	+	+
	FTT_0345	Inner Membrane	+	+
	FTT_0398c	Inner Membrane	+	+
	FTT_0399c	Inner Membrane	+	+
	FTT_0404	Inner Membrane	+	+
	FTT_0715	Inner Membrane	+	-
	FTT_0879	Inner Membrane	+	+
	FTT_0891	Periplasm	+	+
	FTT_1736c	Inner Membrane	+	-
**Secretion systems**				
	FTT_0068	Secreted	+	+
	FTT_0221	Secreted	+	-
	FTT_0484	Secreted	+	+
	FTT_0611c	Secreted	+	Φ
	FTT_0721	Secreted	+	Φ
	FTT_1095	Type 1 System	+	+

FTT_1258	Type 1 System	+	-
FTT_1269c	Secreted DNA K	+	+
FTT_1344	Type 6 System	+	Φ
FTT_1345	Type 6 System	+	Φ
FTT_1346	Type 6 System	+	Φ
FTT_1347	Type 6 System	+	+
FTT_1348	Type 6 System	+	-
FTT_1349	Type 6 System	+	+
FTT_1350	Type 6 System	+	-
FTT_1351	Type 6 System	+	-
FTT_1352	Type 6 System	+	-
FTT_1353	Type 6 System	+	-
FTT_1354	Type 6 System	+	Φ
FTT_1355	Type 6 System	+	+
FTT_1356c	Type 6 System	+	-
FTT_1357c	Type 6 System	+	-
FTT_1358c	Type 6 System	+	-
FTT_1359c	Type 6 System	+	-
FTT_1360c	Type 6 System	+	-
FTT_1361c	Secreted	Φ	+
FTT_1441	Secreted	+	Φ
FTT_1695	Secreted	+	+
FTT_1696	Secreted	+	+
FTT_1724c	Type 1 System	+	+

[a]Based on Meibom et al. 2010 and Rowe et al. 2015.

[b]From NC_006570.2

[c]Gene present (+), absent (-), pseudogenized (Φ).

Table S3. Summary of read assignments and coverage depth of FLE-Am contigs.

Contig	Length (bp)	Reads Mapped	Coverage Depth
1	454,721	355,833	75.88
2	386,665	341,266	84.3
3	269,887	223,506	77.35
4	250,614	197,334	76.11
5	89,993	77,907	83.26
6	88,538	76,923	84.96
7	15,837	12,909	77.21

Table S4. Genes in FLE-Am not present in *F. tularensis* SCHU-S4.

Locus tag	Function[a]
AS144_06750	23S rRNA (pseudouridine(1915)-N(3))-methyltransferase RlmH
AS144_00955	amino acid transporter
AS144_05620	anhydro-N-acetylmuramic acid kinase
AS144_07080	cytochrome BD oxidase subunit I
AS144_02345	D-tyrosyl-tRNA(Tyr) deacylase
AS144_01600	death-on-curing protein
AS144_01245	deoxyribodipyrimidine photolyase
AS144_05170	flippase
AS144_06210	GDP-fucose synthetase
AS144_06215	GDP-mannose 4,6-dehydratase
AS144_01825	glutaredoxin
AS144_05350	glycosyl transferase
AS144_06075	glycosyl transferase
AS144_06085	glycosyl transferase
AS144_06090	glycosyl transferase
AS144_06095	glycosyl transferase
AS144_06080	glycosyl transferase family 1
AS144_00750	hypothetical protein
AS144_01240	hypothetical protein
AS144_01260	hypothetical protein
AS144_01390	hypothetical protein
AS144_01595	hypothetical protein
AS144_01615	hypothetical protein
AS144_01620	hypothetical protein
AS144_01625	hypothetical protein
AS144_01630	hypothetical protein
AS144_01640	hypothetical protein
AS144_01670	hypothetical protein
AS144_01780	hypothetical protein
AS144_01795	hypothetical protein
AS144_01815	hypothetical protein
AS144_01840	hypothetical protein
AS144_01885	hypothetical protein
AS144_01950	hypothetical protein
AS144_01955	hypothetical protein
AS144_01960	hypothetical protein
AS144_01965	hypothetical protein
AS144_01970	hypothetical protein
AS144_01975	hypothetical protein
AS144_02120	hypothetical protein
AS144_02125	hypothetical protein

AS144_02130	hypothetical protein
AS144_02135	hypothetical protein
AS144_02140	hypothetical protein
AS144_02145	hypothetical protein
AS144_02180	hypothetical protein
AS144_02260	hypothetical protein
AS144_02270	hypothetical protein
AS144_02310	hypothetical protein
AS144_02625	hypothetical protein
AS144_02710	hypothetical protein
AS144_02775	hypothetical protein
AS144_02785	hypothetical protein
AS144_02865	hypothetical protein
AS144_02975	hypothetical protein
AS144_02980	hypothetical protein
AS144_02985	hypothetical protein
AS144_02990	hypothetical protein
AS144_02995	hypothetical protein
AS144_03000	hypothetical protein
AS144_03005	hypothetical protein
AS144_03010	hypothetical protein
AS144_03015	hypothetical protein
AS144_03020	hypothetical protein
AS144_03055	hypothetical protein
AS144_03610	hypothetical protein
AS144_03635	hypothetical protein
AS144_03715	hypothetical protein
AS144_03720	hypothetical protein
AS144_03790	hypothetical protein
AS144_03895	hypothetical protein
AS144_04105	hypothetical protein
AS144_04135	hypothetical protein
AS144_03505	hypothetical protein
AS144_04210	hypothetical protein
AS144_04435	hypothetical protein
AS144_04595	hypothetical protein
AS144_04745	hypothetical protein
AS144_04750	hypothetical protein
AS144_04800	hypothetical protein
AS144_04840	hypothetical protein
AS144_04870	hypothetical protein
AS144_04875	hypothetical protein
AS144_04885	hypothetical protein
AS144_04925	hypothetical protein

AS144_05115	hypothetical protein
AS144_05120	hypothetical protein
AS144_05125	hypothetical protein
AS144_05130	hypothetical protein
AS144_05135	hypothetical protein
AS144_05145	hypothetical protein
AS144_05150	hypothetical protein
AS144_05175	hypothetical protein
AS144_05180	hypothetical protein
AS144_05220	hypothetical protein
AS144_05235	hypothetical protein
AS144_04265	hypothetical protein
AS144_05575	hypothetical protein
AS144_05590	hypothetical protein
AS144_05595	hypothetical protein
AS144_05715	hypothetical protein
AS144_05770	hypothetical protein
AS144_5815	hypothetical protein
AS144_05820	hypothetical protein
AS144_05825	hypothetical protein
AS144_05830	hypothetical protein
AS144_05990	hypothetical protein
AS144_06065	hypothetical protein
AS144_06100	hypothetical protein
AS144_06105	hypothetical protein
AS144_06110	hypothetical protein
AS144_06120	hypothetical protein
AS144_06125	hypothetical protein
AS144_06130	hypothetical protein
AS144_06135	hypothetical protein
AS144_06140	hypothetical protein
AS144_06145	hypothetical protein
AS144_06150	hypothetical protein
AS144_06155	hypothetical protein
AS144_06235	hypothetical protein
AS144_06240	hypothetical protein
AS144_06245	hypothetical protein
AS144_06250	hypothetical protein
AS144_06315	hypothetical protein
AS144_06435	hypothetical protein
AS144_06545	hypothetical protein
AS144_06550	hypothetical protein
AS144_06970	hypothetical protein
AS144_07240	hypothetical protein

AS144_07250	hypothetical protein
AS144_07260	hypothetical protein
AS144_07390	hypothetical protein
AS144_07160	hypothetical protein
AS144_07545	hypothetical protein
AS144_07550	hypothetical protein
AS144_07555	hypothetical protein
AS144_07560	hypothetical protein
AS144_07565	hypothetical protein
AS144_07585	hypothetical protein
AS144_06220	mannose-1-phosphate guanylyltransferase/mannose-6-phosphate isomerase
AS144_03850	mannose-6-phosphate isomerase
AS144_05355	mannosyltransferase
AS144_06935	mechanosensitive ion channel protein MscS
AS144_04605	MFS sugar transporter
AS144_02935	MFS transporter
AS144_04835	Nif3-like dinuclear metal center hexameric protein
AS144_01865	phage infection protein
AS144_05360	polymerase
AS144_02335	prephenate dehydrogenase
AS144_06175	pseudaminic acid cytidylyltransferase
AS144_06165	pseudaminic acid synthase
AS144_06045	recombination factor protein RarA
AS144_05165	SAM-dependent methyltransferase
AS144_02895	serine permease
AS144_06115	teichoic acid ABC transporter permease
AS144_05710	transporter
AS144_06180	UDP-4-amino-4,6-dideoxy-N-acetyl-beta-L-altrosamine transaminase
AS144_06185	UDP-N-acetylglucosamine 4,6-dehydratase (inverting)

[a]Function assigned by NCBI Prokaryotic Genome Annotation Pipeline.
